# Longitudinal associations between ambient PM_2.5_ exposure and lipid levels in two Indian cities

**DOI:** 10.1097/EE9.0000000000000295

**Published:** 2024-04-04

**Authors:** Kritika Anand, Gagandeep Kaur Walia, Siddhartha Mandal, Jyothi S. Menon, Ruby Gupta, Nikhil Tandon, K. M. Venkat Narayan, Mohammed K. Ali, Viswanathan Mohan, Joel D. Schwartz, Dorairaj Prabhakaran

**Affiliations:** a Centre for Chronic Disease Control, New Delhi, India; b Public Health Foundation of India, Gurugram, India; c All India Institute of Medical Sciences, New Delhi, India; d Emory Global Diabetes Research Center of the Woodruff Health Sciences Center, Atlanta, Georgia; e Rollins School of Public Health, Emory University, Atlanta, Georgia; f Madras Diabetes Research Foundation (MDRF), Chennai, India; g Harvard T.H. Chan School of Public Health, Harvard University, Boston, Massachusetts

**Keywords:** India, LMIC, Particulate matter, Lipids, Ambient air pollution, Cardiometabolic diseases

## Abstract

**Background:**

Exposure to ambient PM_2.5_ is known to affect lipid metabolism through systemic inflammation and oxidative stress. Evidence from developing countries, such as India with high levels of ambient PM_2.5_ and distinct lipid profiles, is sparse.

**Methods:**

Longitudinal nonlinear mixed-effects analysis was conducted on >10,000 participants of Centre for cArdiometabolic Risk Reduction in South Asia (CARRS) cohort in Chennai and Delhi, India. We examined associations between 1-month and 1-year average ambient PM_2.5_ exposure derived from the spatiotemporal model and lipid levels (total cholesterol [TC], triglycerides [TRIG], high-density lipoprotein cholesterol [HDL-C], and low-density lipoprotein cholesterol [LDL-C]) measured longitudinally, adjusting for residential and neighborhood-level confounders.

**Results:**

The mean annual exposure in Chennai and Delhi was 40 and 102 μg/m^3^ respectively. Elevated ambient PM_2.5_ levels were associated with an increase in LDL-C and TC at levels up to 100 µg/m^3^ in both cities and beyond 125 µg/m^3^ in Delhi. TRIG levels in Chennai increased until 40 µg/m^3^ for both short- and long-term exposures, then stabilized or declined, while in Delhi, there was a consistent rise with increasing annual exposures. HDL-C showed an increase in both cities against monthly average exposure. HDL-C decreased slightly in Chennai with an increase in long-term exposure, whereas it decreased beyond 130 µg/m^3^ in Delhi.

**Conclusion:**

These findings demonstrate diverse associations between a wide range of ambient PM_2.5_ and lipid levels in an understudied South Asian population. Further research is needed to establish causality and develop targeted interventions to mitigate the impact of air pollution on lipid metabolism and cardiovascular health.

**What this study adds:**

The relationship between exposure to PM_2.5_ and lipid levels remains unexplored in developing countries, such as India, having high levels of air pollution and predilection to atherogenic dyslipidemia. The existing literature on this relationship has been inconsistent. Our study fills this gap by investigating the associations using robust estimates of ambient PM_2.5_ exposure, covering a wide range of concentrations in a longitudinal study within two major metropolitan Indian cities. We found significant associations between ambient PM_2.5_ exposure and increased levels of atherogenic lipoproteins (low-density lipoprotein cholesterol, total cholesterol, and triglycerides), coupled with lower levels of protective lipoproteins (high-density lipoprotein cholesterol), with variations between the two cities in India.

## Introduction

Air pollution is a global environmental health issue, as the majority of the world’s population resides in places where air quality levels exceed World Health Organization standards.^1^ Ambient air pollution is among the leading risk factors for mortality worldwide and is ranked among the topmost risk factors for both mortality and morbidity in India.^2^ There is a paucity of evidence on the long-term cardiometabolic health impacts of air pollution exposure from low- and middle-income countries (LMIC). A systematic review from LMIC suggests that the majority of the evidence on the effect of ambient PM_2.5_ exposure on cardiometabolic diseases is coming from China (~65% of studies included in the systematic review).^3^ Briefly, in this systematic review from LMIC, the effect estimates ranged from 0.24% to 6.11% increase per 10 μg/m^3^ increase in PM_2.5_ for cardiometabolic disorders such as hypertension, type 2 diabetes, and cardiovascular (CVD) mortality.^3^ Further, it was reported that CVD-related hospitalizations and emergency room visits also increased by 0.3% to 19.6% per 10 μg/m^3^ increase in PM_2.5_.^3^

Altered lipid levels are suggested as potential mediators between exposure to air pollution and cardiovascular disorders through oxidative stress and subsequent systemic inflammatory response.^4–7^ Indians are specifically vulnerable to atherogenic dyslipidemia, which is characterized by lower high-density lipoprotein cholesterol (HDL-C) and higher triglycerides and low-density lipoprotein cholesterol (LDL-C) levels.^8^ The prevalence of elevated LDL-C levels has been reported in approximately 60% of Indians, high triglycerides (TRIG) in as many as 42.6%, and low HDL-C levels in 56%.^9,10^ In the last few years, studies have examined exposure to ambient air pollution, particularly PM_2.5_, and lipid levels,^11–15^ but systematic reviews and meta-analyses^4^ show that the results have been inconsistent, which may be attributed to smaller sample sizes, cross-sectional designs, varying levels of air pollution concentrations, mixtures of pollutants, heterogeneous methods of exposure assessment, and employment of different statistical methods. No such study has been reported from India which has hazardous levels of PM_2.5_ levels^16^ and a relatively distinct lipid profile^8^ than White population and developed countries. Moreover, there is a huge variation in air pollution levels among Indian cities due to seasonal and topological differences.

At the National Institutes of Health (NIH)-funded Global Environmental and Occupational Health (GEOHealth) Hub in India, our team is utilizing existing cohort studies to generate evidence for cardiovascular health impacts of exposure to ambient PM_2.5_ in Indian cities.^17–19^ This study aimed to examine the associations of short-term (monthly) and long-term (annual) exposure to PM_2.5_ with longitudinally measured serum lipid levels (total cholesterol [TC], TRIG, HDL-C, and LDL-C) in Chennai and Delhi, two metropolitan cities of south and north India, respectively.

## Materials and methods

### Study population

We analyzed existing health data from the Centre for cArdiometabolic Risk Reduction in South Asia (CARRS) cohort, which involved a baseline survey followed by repeat assessments in subsequent years with an overall response rate of approximately 85%.^20^ In Delhi, we saw 75% response rate at follow-up 2 and an impressive 98% at follow-up 4. In contrast, Chennai showed 80% at follow-up 2, dropping to 60% at follow-up 4. The details of the cohort are described previously in Kondal et al.^21^ Briefly, a population-based, multistage, cluster random sampling design based on local administrative boundaries was used to recruit adult men and women to be representative of each city. Pregnant women and seriously ill individuals were excluded from the recruitment. Wards were the primary sampling units, which are administrative units that vary largely in size in different states and cities according to the population density. In 2011, Delhi city had 289 wards (total city area of 1,461 km^2^) and Chennai city had 155 wards (total city area: 172 km^2^). Twenty wards were randomly selected from urban districts. Five Census Enumeration Blocks (CEBs) were randomly selected from each of the 20 randomly selected wards to get 100 CEBs from Chennai and Delhi. Finally, 20 households were selected per CEB and two eligible participants, one man and one woman, aged 20 years or older, were selected from each household based on the “Kish method,” which has been used in the World Health Organization’s STEPS surveys.^22^ Census boundaries and population distribution were used to develop the sampling frame for wards and CEBs, and field staff enumerated households within CEBs to ensure up-to-date household maps and adequate coverage of the target population. Household mapping for each of the newly selected CEBs was conducted to establish the sampling frame for random selection of households and participants.

**Figure 1.**
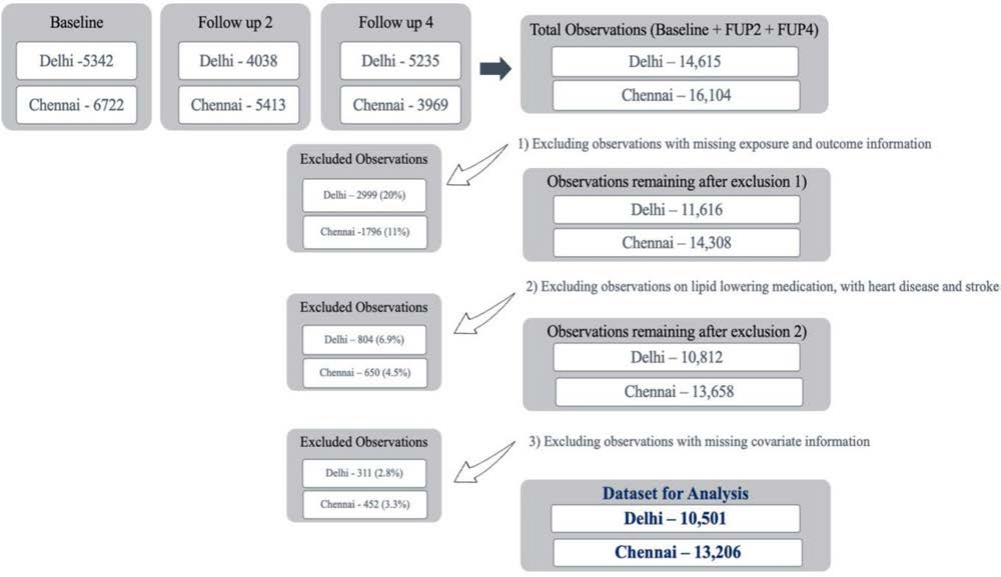
Flowchart detailing inclusion of participants in the study.

CARRS participants were phenotyped for a range of cardiovascular risk factors during the baseline survey.^23^ Thereafter, every year, these participants were followed for CVD events and additionally for detailed lifestyle factors and physical examinations, and every alternate year, for biological samples. The data on the geocoded residence of the participants and how long they have lived at their present location provided an excellent opportunity to estimate air pollution exposure levels at their residence. For the present analyses, we utilized the information from the baseline survey (2010 to 2012), second follow-up (2013 to 2014), and fourth follow-up (2016 to 2017) as the detailed lipid profiling was done in these waves of data collection along with other associated cardiovascular risk factors. Participants without geocoded locations and missing lipid levels at any follow-up were excluded from the analysis (17% in Delhi and 11% in Chennai). Participants included in this analyses did not change their residential location between follow-ups. We also excluded participants who reported statin medication at any time point, participants who had a history of any heart disease or stroke (3.6% in Delhi and 2.6% in Chennai), and participants with missing information on important confounders (3% in both cities). Participants who were on statins at baseline were completely removed from the analysis, whereas participants who initiated statin during the follow-ups or had missing information in only one follow-up were not completely removed. They contributed to the other follow-ups when they met the inclusion criteria (e.g., if statin was initiated in the fourth follow-up, their information until second follow-up was included in the analysis). Figure 1 describes the complete process of sampling for the present analyses.

The CARRS participants provided informed written consent to utilize their deidentified phenotype data and biological samples for future studies and publish the research findings. The GEOHealth Hub Grant was reviewed and approved by the Institutional Ethics Committees of Public Health Foundation of India (Ref. No. TRC-IEC.264.2/15), Centre for Chronic Disease Control (Ref. No. CCDC_IEC_10_2017), Madras Diabetes Research Foundation, and All India Institute of Medical Sciences (Ref. No. IEC/NP-401/09.10.2015).

### Exposure assessment

Exposure to residential-level ambient PM_2.5_ was assessed using retrospective daily average PM_2.5_ predictions at 1 × 1 km grids from a hybrid spatiotemporal model, by assigning the CARRS households to their respective grids. The details of this exposure model have been described previously.^24^ In summary, this was done utilizing satellite data, land use variables, meteorological variables, and population density to build the model using ensemble averaging approaches. The overall prediction accuracy of the model was 80% over the study period.^24^ We used 1-month (short-term) and 1-year (long-term) average exposures for each participant starting from the day before the blood sample collection for the lipid profiling at each follow-up. We will be using short term for the 1-month model and long term for the 1-year model going forward.

### Outcomes

We used data on lipid levels (total cholesterol, triglycerides, HDL-C, and LDL-C) at the baseline, second, and fourth follow-up of the CARRS cohort as continuous measures. Details of the lipid profiling of the participants have been described previously.^23^ Briefly, fasting blood samples were collected from the participants after having a preinformed written consent and the time of the last meal was recorded. Serum HDL-C was estimated directly by an elimination method^25^; total cholesterol was estimated by an enzymatic endpoint method; and triglycerides by GPO-PAP method.^26^ LDL-C level was estimated using standard Friedewald-Fredrickson formula.^27^ As LDL-C was estimated using standard equations based on measured triglycerides and HDL-C, we excluded participants with negative LDL-C values and triglyceride level >400 mg/dl for LDL-C analysis in this study.^28^

### Confounders and covariates

The details of phenotyping for all the other physical measures including anthropometry and questionnaire-based information on medical history for cardiovascular diseases and lifestyle factors have been described in previous studies.^21,23^ Detailed structured questionnaires were administered to collect information on sociodemographic and lifestyle factors including education, occupation, dietary patterns, smoking (current, former, and never), and alcohol consumption (frequency). International Physical Activity Questionnaire (IPAQ) short was used to collect data on physical activity and metabolic equivalence of task scores per day were estimated following standard procedures.^29^ Standard anthropometric procedures were followed to measure body weight, height, and circumferences. Physical activity, body mass index (BMI), and waist-to-hip ratio (WHR) had missing data for follow-ups, hence we used baseline measures as a proxy for overall health or health behavior. Although some data on dietary pattern were available (with less variation within the cohort), a detailed food frequency questionnaire was not used to collect the dietary intake of the participants. Temperature was obtained from daily average global climate reanalysis data provided by the European Centre for Medium-Range Weather Forecasts (ECMWF) at a spatial resolution of 0.125 degrees.^30^ The Normalized Difference Vegetation Index (NDVI) data were sourced from the moderate resolution imaging spectroradiometer data.^31^ We calculated the distance from major roads and shore in geographic information system analysis software by utilizing information from Open Street maps. Socioeconomic status at a neighborhood level was calculated using ward-wise variables from the Census of India House listing and Housing Census data, 2011, which includes variables indicating the quality of households (condition of census house, drinking water source, lighting, facilities, and fuel use) and household assets (banking and household assets such as vehicle ownership, television, and internet).^32^ We performed principal component analysis on these variables to come up with a total score (poorest represented by lower score and wealthiest represented by highest score). The socioeconomics status (SES) score was found to range from −13.4 to 8.3, where a value less than −1.35 belongs to tertile 1 (poorest category), −1.34 to 1.4 falls in tertile 2 (middle category), and >1.4 falls in tertile 3 (wealthiest).

Our confounders include 1-month average temperature (only for monthly exposure models), neighborhood-level socioeconomic status score (based on household quality and assets at a census tract level), road proximity (distance of the residence from a major road), shore proximity (distance of the residence from the shore in Chennai city), calendar time to account for long-term trends (based on the day of blood sample measurement), and the NDVI averaged for 6 months before lipid measurement (indicator of greenness around the residence). Temperature and all the residential factors including neighborhood SES, NDVI, and proximity to road and shore affect both neighborhood ambient PM_2.5_ levels (influencing greenness and traffic in that area) and lipids (influencing dietary choices due to the specific lifestyle, SES of the area, and physical activity due to access to amenities such as major parks and walkability). We did not adjust for individual variables such as age, smoking, and alcohol intake because these lifestyle factors only influence the lipid levels but are not associated with residential PM_2.5_ levels. Relationship of different covariates with ambient PM_2.5_ exposure and the lipid levels are explained using a directed acyclic graph included in the Supplementary Material (Figure S1; http://links.lww.com/EE/A271).

### Statistical analysis

The primary analysis was conducted to examine the association of 1-month average and 1-year average PM_2.5_ exposure with the lipid levels (HDL-C, LDL-C, triglycerides, and total cholesterol) over time. We used random intercept generalized additive models (GAM) to introduce flexibility into the time-varying exposure-response relationship while accounting for within-individual variations/correlation in a longitudinal study. We adjusted for the previously mentioned confounders as linear terms in the models. Penalized splines for PM_2.5_ were used to avoid overfitting of the curve and random intercepts were included for each individual and wards (area of residence) to account for within-participant correlation and spatial clustering effect, respectively. We used inverse probability weighting to correct for bias arising from loss to follow-up in cohort studies. We estimated the probability of continuing in the study based on baseline covariates of the participants. For each city, we predicted how likely someone is to drop out at each follow-up using factors such as age, gender, education, and health status, at baseline. These predictions were used to adjust the weight of each participant’s data in the analysis such that participants who were more likely to drop off were up weighted to make the population similar to the study population at baseline.^33^ The nonlinear spline curve for PM_2.5_ from the random intercept GAM model can be interpreted as the average change in outcome associated with changes in exposure over time while accounting for individual-level differences and spatial clustering after adjusting for potential confounders. The residuals of the models were examined to determine if log transformations were required for outcomes. Effect modification of age, sex, BMI, WHR, and physical activity (computed at baseline using total metabolic scores) on the association between ambient PM_2.5_ exposure and lipid levels was assessed using nonlinear spline interactions. The effect modification was assessed based on a likelihood ratio test between the nonlinear regression models with and without effect modifiers.

## Results

The demographic characteristics of participants are presented in Table 1. The median age of participants at baseline was 44 years (SD = 12.8 years) in Delhi and 40 years (SD = 12.2 years) in Chennai with 51% to 55% of the participants being females. One in five participants reported themselves as alcohol consumers and 20% were current smokers at baseline. The distribution of BMI was similar in the two cities with half of the participants being obese or overweight. WHR >0.85 was seen in 62% of the participants in Chennai, compared with 72% in Delhi. The median monthly temperature before lipid measurements ranged around 24 to 28 °C in both cities across follow-ups. Median 6-month average NDVI also remained the same (0.31 to 0.33) for Chennai residents across follow-ups but varied between 0.22 and 0.27 for Delhi during the 7-year study period. Participants from Chennai lived closer to major roads than participants in Delhi and lived on an average 4.5 km from the shore. The median SES score for Chennai was −0.52 (interquartile range [IQR]: −1.72 to 2.78) and Delhi was 0.36 (−1.49 to 1.99). The SES scores of the two cities cannot be directly compared as they are calculated relative to the study population of the respective cities.

**Table 1.** Descriptive characteristics of Centre for cArdiometabolic Risk Reduction in South Asia (CARRS) cohort for the study period (2010–2017)

Variables	Chennai			Delhi		
	Baseline	Follow-up 2	Follow-up 4	Baseline	Follow-up 2	Follow-up 4
Total study participants	6,055	3,980	3,171	4,528	3,291	2,682
Individual characteristics						
Female; n (%)	3,321 (54.8)	2,302 (57.8)	1,837 (57.9)	2,305 (50.9)	1,730 (52.6)	1,431 (53.4)
Age (years); median (IQR)	39.9 (31.7 to 49.3)	42.14 (34.5 to 51.2)	44.22 (37.1 to 53.1)	43.74 (34.8 to 53.7)	45.89 (37.8 to 54.8)	47.95 (40.2 to 56.2)
Years of education (years); median (IQR)	8 (5 to 10)		9 (5 to 10)	10 (5 to 15)	-	10 (5 to 15)
Current tobacco consumption (any form); n (%): yes	1,188 (19.6)	657 (16.5)	543 (17.1)	1,007 (22.2)	654 (19.9)	529 (19.7)
Current alcohol consumption; n (%): yes	1,280 (21.9)	662 (16.6)	551 (17.4)	768 (17)	550 (16.7)	508 (18.9)
BMI (imputed at baseline) categories at baseline; n (%)						
Normal (<25 kg/m^2^)	2,999 (49.5)			2,184 (48.2)		
Overweight (≥25 and <30 kg/m^2^)	2,099 (34.7)			1,468 (32.4)		
Obese (≥30 kg/m^2^)	957 (15.8)			876 (19.4)		
WHR categories at baseline; n (%)						
<0.85	2,287 (37.8)			1,207 (26.3)		
0.85 to 0.95	2,356 (38.9)			1,812 (39.5)		
>0.95	1,412 (23.3)			1,509 (33.3)		
Physical activity at baseline (based on short IPAQ)						
Quartile 1	1,339			972		
Quartile 2	1,528			1,065		
Quartile 3	1,934			1,159		
Quartile 4	1,129			1,203		
Residential characteristics						
Temperature (°C); median (IQR)	28.43 (27.9 to 29.1)	27.63 (24.8 to 28.2)	28.3 (27.9 to 29.4)	27.04 (19.2 to 29.8)	23.89 (17.6 to 27.1)	27.50 (25.1 to 30.5)
6-month average NDVI; median (IQR)	0.31 (0.28 to 0.36)	0.33 (0.29 to 0.37)	0.32 (0.29 to 0.36)	0.22 (0.18 to 0.28)	0.27 (0.23 to 0.32)	0.22 (0.19 to 0.27)
Neighborhood SES score; median (IQR)	−0.52 (−1.72 to 2.78)	-	-	0.36 (−1.49 to 1.99)	-	-
Proximity to the shore (m); median (IQR)	4,404.6 (1,919.2 to 5,977.16)	-	-	-	-	-
Proximity to major road (m); median (IQR)	11.51 (7.1 to 30.7)	-	-	159.8 (60.7 to 263.4)	-	-
Lipids levels						
HDL-C (mg/dl); median (IQR)	41 (36 to 47)	40 (35 to 46)	40 (34 to 45)	45 (38 to 53)	40 (34 to 48)	40.5 (34.4 to 47.9)
LDL-C (mg/dl); median (IQR)	111.4 (92 to 132)	112 (91.6 to 132.2)	110.2 (91.6 to 130.8)	105.4 (83.7 to 126.85)	110.2 (90 to 133)	105.2 (86.28 to 127.1)
Total cholesterol (mg/dl); median (IQR)	180 (157 to 205)	181 (157 to 205)	179 (158 to 204)	180 (154 to 205.5)	180 (156 to 207)	176.6 (151.9 to 201)
Triglycerides (mg/dl); median (IQR)	118 (86 to 169)	128 (93 to 179)	131 (96 to 180)	123 (92 to 169.25)	125 (95 to 172)	125.5 (93.4 to 174.65)

The trends in lipid levels are presented in Table 1. Overall, both cities had comparable levels of TC at baseline and remained constant over time with a median level of 180 mg/dl (IQR: 154 to 205.5). Median LDL-C at baseline was 111.4 mg/dl (IQR: 92 to 132) in Chennai and 105.4 mg/dl (83.7 to 126.85) in Delhi, the levels of which remained constant in Chennai but first increased then decreased in Delhi over time. In Chennai, median baseline HDL-C was lower (41 mg/dl [IQR: 36 to 47]) and more stable than in Delhi (45 mg/dl [IQR: 38 to 53]) where it decreased over time. In the case of triglycerides, Chennai had a median of 118 mg/dl (IQR: 8 to 169) at baseline, which went up across follow-ups, whereas, in Delhi, median triglycerides was 123 mg/dl (IQR: 92 to 169.25) and remained constant. The distribution of lipids at baseline within quartiles of short-term exposure is given in Table S1; http://links.lww.com/EE/A271. We observed comparable lipid levels across different exposure categories at baseline, with similar patterns for long-term exposure as well. All outcomes except triglycerides were almost normally distributed.

Table 2 provides a summary of ambient PM_2.5_ exposures experienced in the two cities during the study period. Both short- and long-term exposures in Chennai were lower and ranged from 15 to 75 μg/m^3^ compared with very high levels in Delhi with exposures around 30 to 283 μg/m^3^. The mean 1-month exposure in Chennai and Delhi was 37 (SD = 8) μg/m^3^ and 96.43 (SD = 38.37) μg/m^3^, respectively. The mean 1-year exposure in Chennai and Delhi was 40.16 (SD = 4.79) μg/m^3^ and 102.25 (SD = 15.37) μg/m^3^, respectively. Within city variability in exposure across follow-ups was minimal, however, there were a large number of outliers for monthly PM_2.5_ during the fourth follow-up in 2016 (highlighted in Figure S2; http://links.lww.com/EE/A271).

**Table 2.** Exposure summaries across follow-ups for CARRS cohort in the study period

Median PM_2.5_ concentration (µg/m^3^) (25th, 75th percentile)	Delhi		Chennai	
	Monthly average	Annual average	Monthly average	Annual average
Baseline	81.3 (64.2, 99.8)	92.1 (87.6, 95.7)	35.0 (30.8, 43.1)	41.1 (38.7, 43.4)
Follow-up 2	93.1 (67.7, 143.1)	99.3 (92.2, 107.1)	35.7 (32.1, 43.9)	41.5 (38.9, 44.2)
Follow-up 4	91.4 (72.1, 116.9)	121.3 (117.7, 124.8)	33.3 (29.4, 36.7)	36.4 (34.5, 39.9)

**Figure 2.**
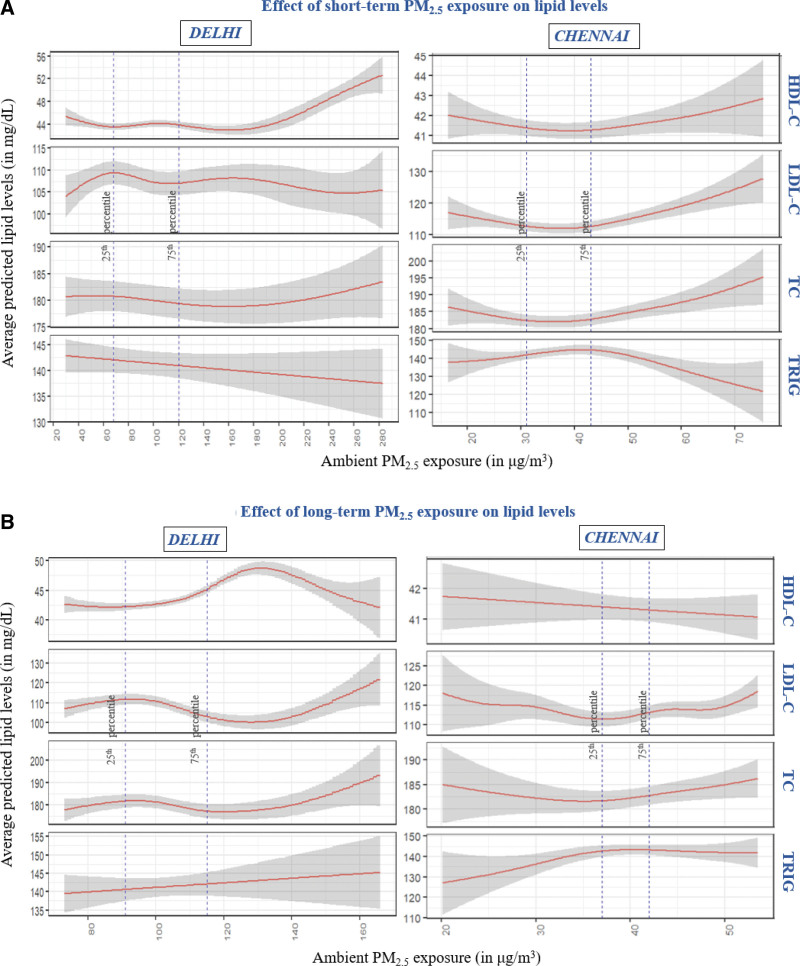
Longitudinal association between short-term (A) and long-term (B) ambient PM_2.5_ exposure and lipid levels in Delhi and Chennai. Shaded areas represent point-wise prediction intervals. The lines depicted correspond to the 25th and 75th percentiles, marking the lower and upper boundaries of the interquartile range. This range encapsulates the associations within the middle 50% of the observations.

The relationship between exposure to ambient PM_2.5_ and lipid levels was found to be nonlinear in our study (Figure 2A,B). We present the predicted levels of the outcome at different exposure levels and the 95% prediction intervals (PI).

(1) HDL-C: Short-term exposure to ambient PM_2.5_ in Delhi showed a positive association with HDL-C in both cities while long-term exposure to PM_2.5_ had no effect on HDL-C below 100 µg/m^3^ in both cities. However, in Delhi, long-term exposures showed linear decrements in HDL-C mostly beyond the 90th percentile (125 µg/m^3^) only.
(2) LDL-C and TC: Short-term exposure to ambient PM_2.5_ was associated with a linear increase in LDL-C in both cities at 40 to 70 µg/m^3^ and concentrations exceeding 70 µg/m^3^ in Delhi, the curve plateaus. In Chennai, the predicted LDL-C was 112.5 mg/dl (95% PI = 110.9, 114) at 30 µg/m^3^ average short-term exposure while it was 119.1 mg/dl (95% PI = 116.3, 121.9) at 60 µg/m^3^. Short-term exposure to ambient particulate matter was not associated with TC in Delhi and showed a similar strong association with short-term exposures in Chennai above 35 µg/m^3^. Long-term exposure to ambient PM_2.5_ was associated with higher levels of both LDL-C and TC in both cities. Chennai showed a deep U-shaped curve with a decrease up to 35 µg/m^3^ and then an increase, whereas, in Delhi, we observed slight increases up to 100 µg/m^3^ and steeper increments at very high exposures above 130 µg/m^3^. A 10 µg/m^3^ increase in long-term PM_2.5_ in Chennai (37 to 47 µg/m^3^) leads to an increase of approximately 2.5 mg/dl (181.7 mg/dl [179.7 to 183.7] to 184.2 mg/dl [181.8 to 186.6]) in TC levels, whereas a 10 µg/m^3^ increase at a steeper slope of Delhi (135 to 145 µg/m^3^) corresponded to 3.3 mg/dl rise (178.6 mg/dl [174.2 to 183.1] to 181.9 mg/dl [176.6 to 187.2]) in TC levels.
(3) TRIG: We ran both log-transformed and non log-transformed models to check for the distribution of residuals. Both models showed comparable results, therefore we present the non log-transformed model for better interpretability. Short-term exposure to ambient PM_2.5_ in Delhi showed slight negative associations with TRIG in both cities. On the other hand, long-term exposure to ambient PM_2.5_ was associated with an increase in TRIG in both cities, with a linear increase in Delhi, however, an increase and then plateauing in Chennai (above 40 µg/m^3^).

**Figure 3.**
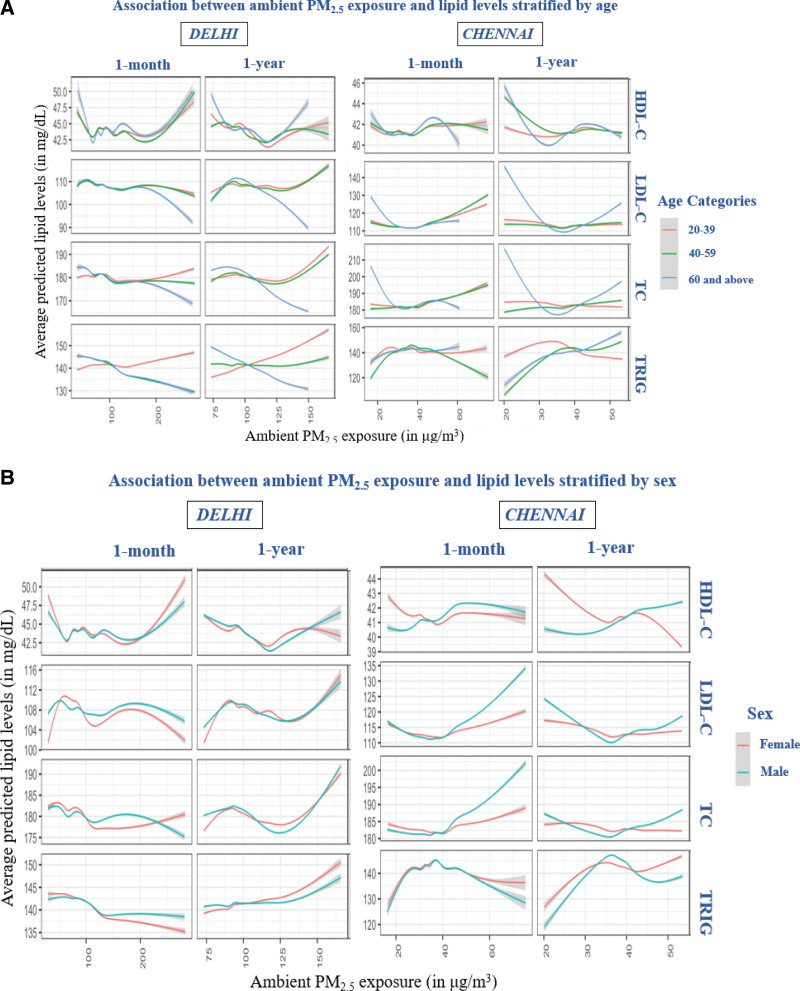
Association between exposure to short-term (A) and long-term (B) ambient PM_2.5_ and lipid levels stratified by effect modifier. Shaded areas represent point-wise prediction intervals.

We observed significant effect modification by age on the association between ambient PM_2.5_ and HDL-C, LDL-C, TC, and TRIG (Figure 3A). In Delhi, the detrimental effects of air pollution (increase in LDL-C, TC, and TRIG) were observed to be greater in young individuals compared to the elderly (≥60 years), for both short- and long-term exposures. In Chennai, the impact of short-term exposure on young individuals was greater; however, the elderly population experienced a greater impact from long-term exposure to air pollution. In Chennai, differences in association between air pollution and lipids were also observed between males and females (Figure 3B). Males showed more substantial increases in LDL-C and TC in response to both short- and long-term exposures to air pollution. In contrast, females in Chennai demonstrated lower HDL-C levels and higher levels of TRIG with an increase in air pollution exposure. Although the differences were less pronounced in Delhi, a similar pattern was observed in Chennai. We did not observe any clear patterns in effect modification for other physical activity, BMI, and WHR (Figure S3; http://links.lww.com/EE/A271).

## Discussion

Our study aimed to investigate the longitudinal association of short-term (1-month average) and long-term (1-year average) exposure to ambient PM_2.5_ on lipid levels in two Indian cities. Overall, we observed a significant increase in atherogenic lipids (LDL-C, TC, and TRIG) and a decrease in protective lipid (HDL-C) with an increase in both short- and long-term ambient PM_2.5_ exposures. We noted some city-specific differences. In Chennai, exposure to ambient PM_2.5_ was significantly associated with increased LDL-C, TC, and TRIG; however, steeper slopes were seen for short-term exposure. In Delhi, we observed detrimental effects of LDL-C (at levels <100 µg/m^3^) and TRIG against monthly average exposures, while long-term exposure to ambient PM_2.5_ had a pronounced impact on the lipid levels of the residents of Delhi. The steeper curves at the highest quartile of exposure should be interpreted carefully based on the exposure distribution and precision of the estimates.

The notable differences observed in the association results of Delhi and Chennai may be explained by a marked difference in the levels of PM_2.5_ in these two cities in addition to seasonal and lifestyle differences that may contribute to different metabolic profiles. Delhi is the national capital situated in north India within the Indo-Gangetic plain witnessing extremes of summers and winters. In addition to its own pollution sources, land-locked Delhi also receives pollutants from adjoining north Indian states. The wind direction, low speed, and low temperature become unfavorable for the dispersion of pollutants, thus making Delhi more polluted than Chennai. On the other hand, Chennai is a coastal city in southern India that experiences moist summers and does not experience winters, which helps in the dispersion of pollutants. These climatic and topological factors cause the major differences in PM_2.5_ levels. Moreover, there are ethnic and sociocultural differences between the two cities, which affects the metabolism and lipid levels. Lifestyle factors such as dietary patterns that greatly influence the lipid levels, are majorly determined by the climatic factors, dietary choices, and cooking practices specific to different ethnic and geographic populations. Further, our present findings suggest a greater detrimental effect of continuously high exposures leading to a detectable change in lipid levels (greater long-term responses in Delhi compared to more short-term effects on lipids in Chennai). This could be because, in areas with lower levels of air pollution (such as Chennai), the threshold for observing changes in lipid levels might be easily surpassed, which leads to easy detection of increased lipid levels attributable to short-term spikes in air pollution in comparison to detecting short-term impacts in Delhi. The areas with lower levels of air pollution might have a smaller “buffer” before the threshold for detecting increased lipid levels is reached (as the threshold is lower), and people living in these areas are less likely to have adapted to the effects of air pollution. Individuals in Delhi may have adapted to high air pollution levels, which might reduce their sensitivity to short-term fluctuations in ambient PM_2.5_ levels. Concentration–response curves for air pollution and health effects have often shown plateauing of health effects at higher air pollution levels.^34^ Therefore, exposures in Chennai are more in the range where effects are detected, whereas in Delhi we observed effects either at <100 µg/m^3^ or at extremely high exposures (>90th percentile).

In our study, we also observed distinct patterns of air pollution effects on different age groups. In Delhi, the younger population showed a higher susceptibility to air pollution exposure compared to Chennai, where the elderly participants were more affected. Chennai had greater detrimental impacts on the elderly, which might be due to differences in dietary patterns and lifestyle choices among the younger individuals in the two cities. The differences in Delhi might be because elderly participants might have had comorbid conditions and could have been taking medications such as beta blockers and antidiabetic drugs, which could influence their lipid levels and potentially limit their overall exposure to ambient air pollution. Interestingly, we did not observe any differences in the associations between genders in Delhi. In addition to perennial exposure to high PM_2.5_ levels, there could be uniformity in the lifestyle and health care seeking behavior between genders in the city, which might explain similar outcomes. In Chennai, however, males exhibited increased levels of LDL-C and total cholesterol, along with a surge in HDL-C. These findings highlight the complex interplay between air pollution and effect modifiers (such as age, gender, and lifestyle habits), which collectively influence the lipid profiles and susceptibility to the adverse effects of air pollution in different populations. Studies have shown greater impacts of air pollution on the elderly and males, which is similar to our results from Chennai, which is within the range of PM_2.5_ concentrations of these studies.^35,36^ We did not observe effect modification by physical activity, BMI, and WHR in our data, which could be because these were measured only at baseline and might have changed over time (Figure S3; http://links.lww.com/EE/A271). We could not examine effect modification by dietary factors as detailed information on dietary intake (food frequency questionnaire) was not captured and there was minimal variation in the dietary data available for analyses.

The exact biological mechanisms to explain the association between particulate matter exposure and lipid levels are not very clear but the most suggested ones include the oxidative stress and systemic inflammation that affect lipid metabolism.^7^ Altered DNA methylation of candidate genes involved in lipid metabolism induced by air pollution exposure is also suggested to play a mediating role between exposure to PM_2.5_ and altered lipid levels,^37^ thereby, making them ideal targets for therapeutic intervention^38^ to reduce cardiometabolic diseases. The current body of literature on the possible role of ambient air pollution exposure on altered lipid levels is inconsistent.^4^ Kim et al^15^ did not find significant associations between exposure to air pollution and lipid levels in a Korean population. This might be because this study was conducted at a lower PM_2.5_ concentration setting in South Korea and among healthy soldiers that have different physique and metabolic profile than the general population. Study conducted in an Italian birth cohort also showed no associations between ambient air pollution and childhood obesity parameters including total cholesterol and HDL-C.^39^ Nevertheless, studies conducted in United States, Israel, and China provide evidence to suggest a possible link between air pollution exposure and lipid levels,^11,40–42^ where stronger associations of ambient air pollution exposure were found among people with high levels of lipid than those with lower levels of lipids.^11,42^ There is conflicting evidence for different lipid measures as well. Bell et al^43^ in a cross-sectional design found only long-term and no short-term effects between PM_2.5_ and HDL-C. A Chinese quasi-experimental longitudinal study found a significant effect of long-term exposure on total cholesterol and LDL-C but not with triglycerides and HDL-C.^44^ A cohort study from China also reported that long-term exposure to PM_2.5_ was associated with a significant increase in LDL-C, weaker association with TC, and no association with HDL-C and triglycerides.^45^ A study from an elderly population in Taiwan showed that increased 1-year average PM_2.5_ was associated with increased total cholesterol levels at PM_2.5_ concentrations similar to Chennai in our study.^46^ A study conducted in northwestern China among type 2 diabetes patients also looked at nonlinear associations between lipid levels and short-term exposure to SO_2_, NO_2,_ and PM_10_. The authors found J-shaped and inverted U-shaped curves with similar patterns for different lipid parameters (increase in HDL-C, LDL-C, TC, and plateauing of TRIG effects).^47^

To the best of our knowledge, this is the first study conducted in India to demonstrate the impact of monthly and annual exposure to ambient PM_2.5_ on lipid levels. Our study findings align with existing global literature, particularly pertinent to diverse South Asian populations with predilection to atherogenic dyslipidemia encompassing a wide range of ambient PM_2.5_ exposure levels. Moreover, this study includes two metropolitan cities in India, which are distinct in geography, lifestyle factors, and air pollution levels, making our study more generalizable. The findings are based on a large sample size with repeated measurements spanning from 2010 to 2017, hence, providing robust and comprehensive estimates. To address missing covariate data, we have used imputations and inverse probability weighting methods were used to correct for bias from loss to follow-up. Furthermore, exposure assessment was carried out using a highly reliable prediction model at fine spatial-temporal resolution. Although personal monitoring is most ideal for examining the effect of air pollution on an individual’s health outcomes, it is not logistically feasible to personally monitor such a huge number of participants regularly over the years. Exposure assessment at the residential level reduces confounding by individual factors (individual-level variables such as physical activity or occupation do not affect air pollution at a residential level) but may lead to exposure misclassification, which may bias results. We accounted for all the available and possible confounders; however, there is still a possibility of unmeasured confounding due to occupational exposures, noise, and more robust physical activity measurement. Anthropometrics and physical activity data were not reliable for the follow-ups; therefore, we used the baseline data. Unfortunately, we did not have detailed dietary intake information to estimate fat consumption specifically, which greatly influences the lipid levels.

We found that exposure to ambient particulate matter is associated with altered lipid levels in the Indian population. Increase in LDL-C, total cholesterol, and triglycerides and decrease in HDL-C are known risk factors for developing cardiovascular disease; therefore, findings from this study can assist in strengthening evidence and advocate for policy changes to reduce air pollution levels. While our longitudinal study adds to the inadequate evidence from low- and middle-income countries, further studies from regions with varying levels of pollution, geography, ethnicity, and different age groups are required to gain a more nuanced understanding of this relationship and resolve the inconsistencies in the literature.

## Conflicts of interest statement

The authors declare that they have no conflicts of interest with regard to the content of this report.

K.A. was involved in conceptualization, developing methodology, conducting the formal analysis, visualization, and writing the original draft. G.K.W. was involved in conceptualization, developing methodology, and writing the original draft. S.M. and J.D.S. provided methodological and analysis supervision. J.S.M. provided data analysis resources. R.G., N.T., K.M.V.N., M.K.A., V.M., D.P., and J.D.S. contributed to the conceptualization, investigation, resources, and funding acquisition of the CARRS cohort. All authors edited and approved the final version of the manuscript.

## Acknowledgments

We would like to thank the CARRS study participants and the entire GEOHEALTH and CARRS study team from Center for Chronic Disease Control, Public Health Foundation of India, Madras Diabetes Research Foundation, Harvard TH Chan School of Public Health, and Rollins School of Public Health, Emory University for their support in the present work.

## Supplementary Material


