# Supplementary material for: Longitudinal associations between ambient PM2.5 exposure and lipid levels in two Indian cities
Source: Environ Epidemiol. 2024 Apr 4;8(2):e295. doi: 10.1097/EE9.0000000000000295 (PMC11008625; doi:10.1097/EE9.0000000000000295)
Supplement: Supplementary file 1 [file ee9-8-e295-s001.docx]

**Supplementary Material**

**Table S1: Distribution of lipid levels within quartiles of exposure of Centre for cArdiometabolic Risk Reduction in South-Asia (CARRS) cohort at baseline**

| 1-month  Exposure | Chennai | | | | Delhi | | | |
| --- | --- | --- | --- | --- | --- | --- | --- | --- |
|  | HDL-C | LDL-C | TC | TRIG | HDL-C | LDL-C | TC | TRIG |
|  | Mean (IQR) | | | | | | | |
| Quartile 1 | 42.9 (36,48) | 113.11  (91.42,131.95) | 183.12 (158,205) | 135.46  (86,169) | 46.92  (39, 53) | 109.06 (86.21,128.42) | 184.05  (157, 209) | 140.37  (93,169) |
| Quartile 2 | 41.51  (35,47) | 113.51  (92.65,131.15) | 182.05 (156.7,204) | 135.18  (86,165) | 45.92  (37,52) | 106.70  (83.7, 126.6) | 181.36  (154,205) | 143.74  (94,174) |
| Quartile 3 | 41.71  (35,47) | 109.53  (90,128.8) | 178.69 (154,201) | 137.24  (86,173) | 46.61  (37,54) | 105.57  (80.54,126.2) | 179.4  (151, 205) | 136.08  (88,164) |
| Quartile 4 | 41.97  (36,47) | 114.90  (93.62,135.0) | 184.03  (158,208) | 135.79 (85,167) | 45.77  (38,52) | 105.56  (84.47,124.9) | 178.62  (152.6,203) | 136.4  (91,167) |

*Footnotes: Quartiles for Delhi: [29.8,68.1], (68.1,85.2], (85.,120], (120,283]*

*Quartiles for Chennai: [16.3,31.1], (31.1,34.7], (34.7,42.7], (42.7,75.3]*

*HDL-C: High Density Lipoprotein; LDL-C: Low Density Lipoprotein; TC: Total Cholesterol; TRIG: Triglyceride*

**Figure S1: Boxplot of Ambient PM_2.5_ exposure metrics across follow-ups for two cities**


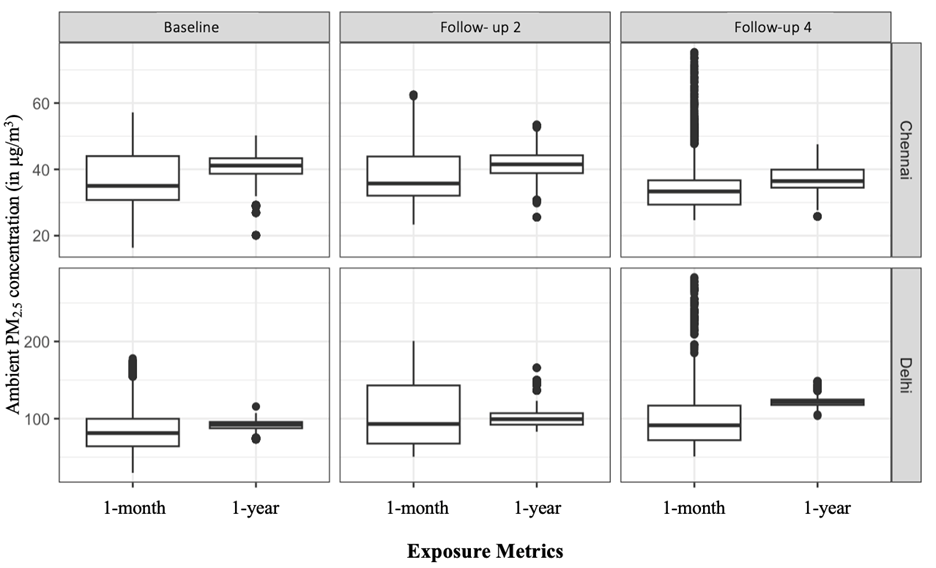


*Footnote: The box of a boxplot starts in the first quartile (25%) and ends in the third (75%), with the horizontal line in bold that represents the median. The horizontal lines outside of the boxes represent the minimum/maximum values excluding outliers, while the dots represent the outliers. Please note the y-axis is different for the two cities.*

**Figure S2: Directed Acyclic Graph for the association between exposure to ambient PM_2.5_ and lipid levels.**


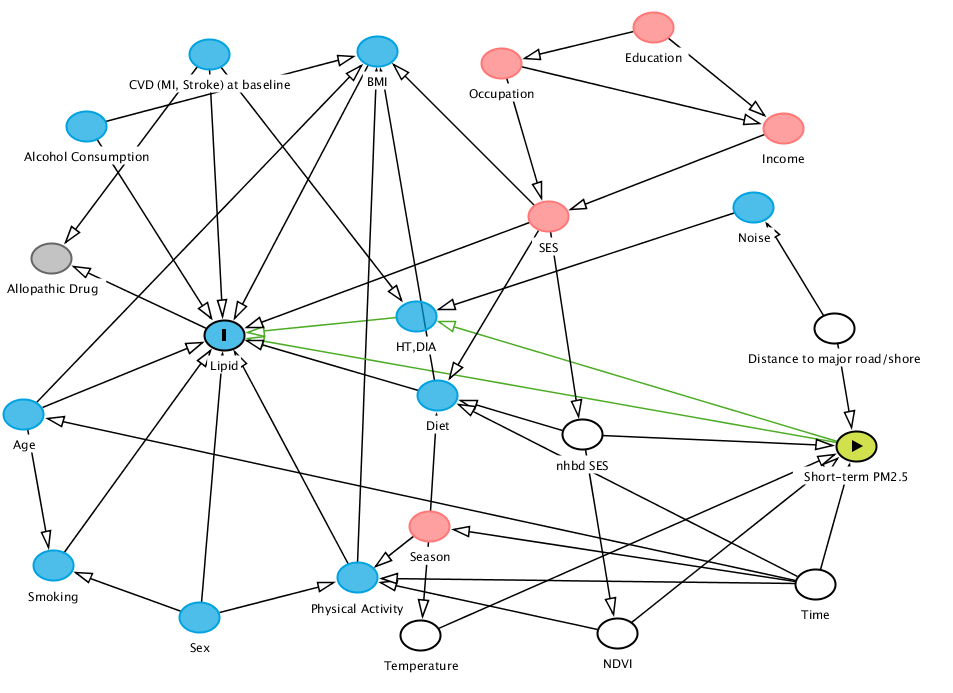


**Footnote:** CVD-cardiovascular disease; MI- Myocardial Infarction; BMI- Body Mass Index. HT- Hypertension; DIA- Diabetes status; SES- Socio-economic status; NDVI – Normalized Difference Vegetation Index; nhbd SES- Neighborhood Socio-economic status. We adjusted for confounders denoted as white circles in the DAG.

**Figure S3: Association between exposure to ambient PM_2.5_ and lipid levels stratified by effect modifier.**


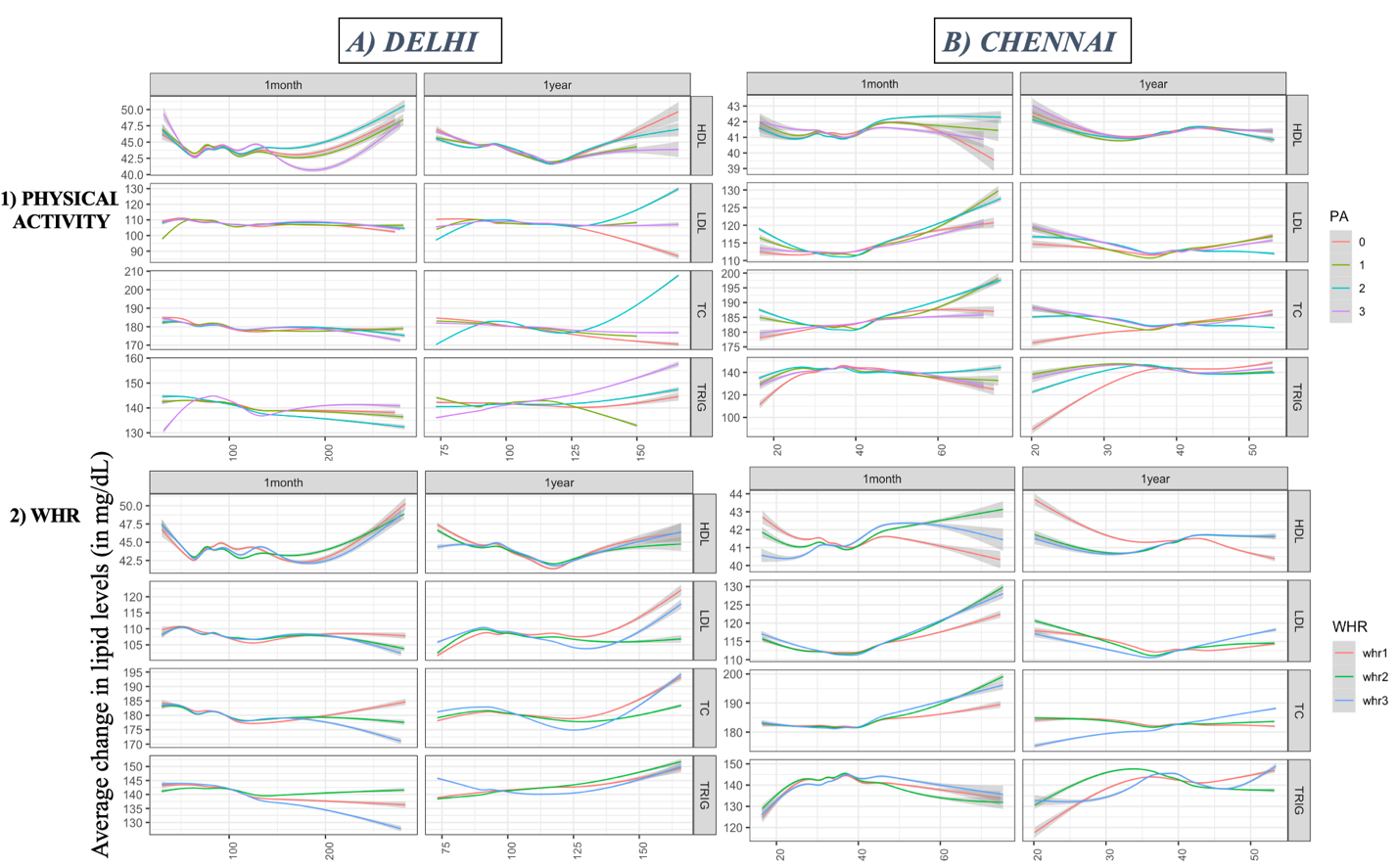

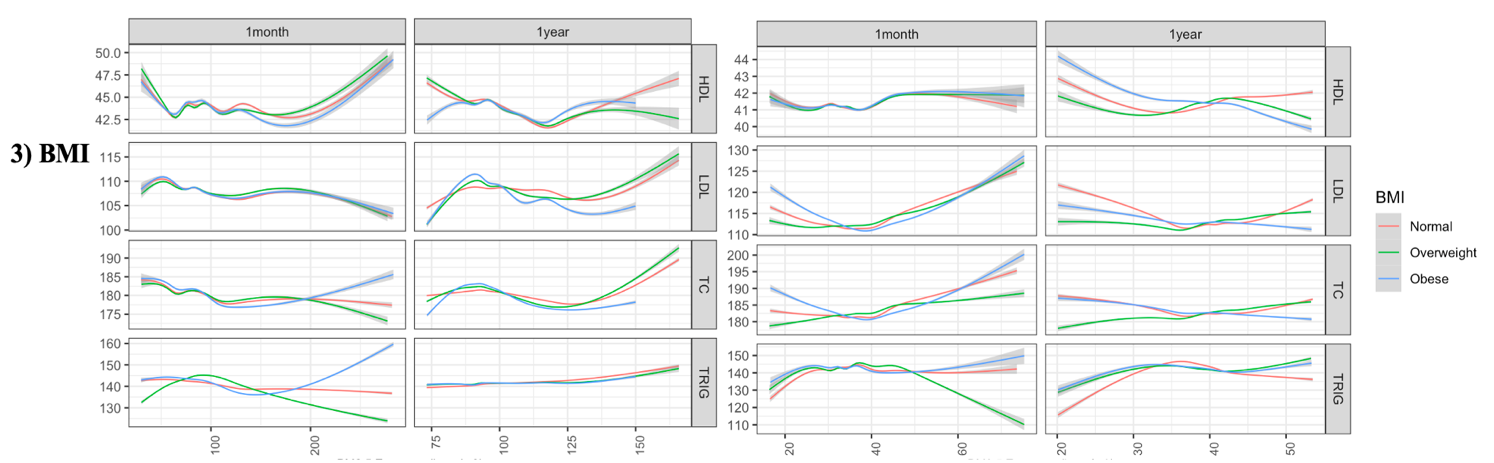


Note: PA: physical activity (category 0 is the lowest and 3 is the highest) ; WHR: Waist to Hip Ratio (category 1 is the lowest and 3 is the highest) ; BMI: Body Mass Index.
